# Complete Genome Sequence of *Herbinix luporum* SD1D, a New Cellulose-Degrading Bacterium Isolated from a Thermophilic Biogas Reactor

**DOI:** 10.1128/genomeA.00687-16

**Published:** 2016-07-21

**Authors:** Daniela E. Koeck, Irena Maus, Daniel Wibberg, Anika Winkler, Vladimir V. Zverlov, Wolfgang Liebl, Alfred Pühler, Wolfgang H. Schwarz, Andreas Schlüter

**Affiliations:** a Institute for Microbiology, Technische Universität München, Freising-Weihenstephan, Germany; b Institute of Molecular Genetics, Russian Academy of Science, Moscow, Russia; c Center for Biotechnology (CeBiTec), Genome Research of Industrial Microorganisms, Bielefeld University, Bielefeld, Germany

## Abstract

A novel cellulolytic bacterial strain was isolated from an industrial-scale biogas plant. The 16S rRNA gene sequence of the strain SD1D showed 96.4% similarity to *Herbinix hemicellulosilytica* T3/55^T^, indicating a novel species within the genus *Herbinix* (family *Lachnospiraceae*). Here, the complete genome sequence of *Herbinix luporum* SD1D is reported.

## GENOME ANNOUNCEMENT

The isolation, characterization, and genome sequencing of new strains are pivotal to link their genetic potential to possible ecological roles in biomass digestion and biogas production (1). Cellulolytic isolates, like those of *Clostridium thermocellum*, *Clostridium cellulosi*, and *Herbinix hemicellulosilytica*, are a promising source for novel biomass-degrading enzymes that convert biomass into sugars, the basis for the production of biofuels, bulk chemicals, and other valuable products (2–4).

The new cellulose-degrading strain SD1D was isolated from a thermophilic biogas plant, as described previously (5). A nucleotide sequence comparison between the isolate SD1D and *H. hemicellulosilytica* T3/55^T^ revealed a 16S rRNA sequence similarity of 96.4%, as well as an average nucleotide identity (ANI) of 85.73%, suggesting strain SD1D as a new species within the genus *Herbinix* (family *Lachnospiraceae*) (6, 7).

*Herbinix luporum* SD1D is able to digest cellulosic and hemicellulosic substrates (filter paper, phosphoric acid swollen cellulose, carboxymethyl cellulose, xylan, and barley). The highest activity was determined to occur on xylan, but in contrast to strain T3/55^T^, SD1D was not able to grow on xylan or xylose. Galactose, glucose, mannose, arabinose, cellobiose, and cellulose are utilized as sole sources of carbon. The major fermentation products are ethanol, acetic acid, and small amounts of propionic acid.

The DNA of *H. luporum* SD1D was extracted and used to construct an 8-kb mate-pair sequencing library (Nextera mate-pair sample preparation kit; Illumina, Inc.), which was sequenced on an Illumina MiSeq system. The sequencing run yielded 1,766,358 reads, accounting for 357,174,077 bases of total sequence information. The obtained sequences were *de novo* assembled (8) using the GS *De Novo* Assembler software (version 2.8; Roche) and resulted in one scaffold comprising 19 contigs. After an *in silico* gap closure approach, a circular chromosome of 2,609,352 bp in size, featuring a G+C content of 35.25%, was established. The software platform GenDB (9) was applied to annotate the *H. luporum* SD1D genome, leading to the identification of 2,362 protein-coding sequences, 53 tRNA genes, and four *rrn* operons.

The SD1D genome was searched for the presence of carbohydrate-active enzymes by applying the Hidden-Markov-Model (HMM)-based carbohydrate-active enzyme annotation database dbCAN (10). In total, 174 genes encode enzymes or modules with predicted activity on carbohydrates that mainly belong to different families of glycoside hydrolases (GH). Among them, four putative cellulosomal genes were identified encoding GH family modules (2× GH9, GH48, and GH11), each attached to a dockerin module. A corresponding scaffoldin (*cipA*) comprising five type 1 cohesin and four carbohydrate-binding modules (CBM_X2) is encoded in the genome of SD1D. In addition, five potential cellulases of the GH family 5 were identified. Except for one endoglucanase gene, all other cellulosomal genes are arranged in one cluster. In contrast, *H. hemicellulosilytica* T3/55^T^ does not possess any putative cellulosomal genes.

The availability of the *H. luporum* SD1D genome sequence provides the genetic basis for the biotechnological exploitation of genome features involved in the thermophilic degradation of lignocellulosic biomass.

### Nucleotide sequence accession numbers.

This whole-genome shotgun project has been deposited in the EMBL/GenBank database (EBI, NCBI) under the accession no. LN879430. The strain is available from the Leibniz Institute German Collection of Microorganisms and Cell Cultures (DSMZ, Braunschweig, Germany) under the accession no. DSM 100831.
